# Donor-recipient age difference, not donor age alone, predicts outcomes in allogeneic hematopoietic stem cell transplantation for aplastic anemia

**DOI:** 10.3389/fimmu.2026.1894494

**Published:** 2026-08-24

**Authors:** Zhengwei Tan, Yuechao Zhao, Huijin Hu, Yu Zhang, Tonglin Hu, Dijiong Wu, Baodong Ye, Wenbin Liu

**Affiliations:** 1 Department of Hematology, The First Affiliated Hospital of Zhejiang Chinese Medical University (Zhejiang Provincial Hospital of Chinese Medicine), Hangzhou, China; 2 The First School of Clinical Medicine, Zhejiang Chinese Medical University, Hangzhou, China

**Keywords:** age difference, aplastic anemia, donor age, donor selection, hematopoietic stem cell transplantation, overall survival

## Abstract

**Background:**

The impact of donor age on outcomes after allogeneic hematopoietic stem cell transplantation (allo-HSCT) for aplastic anemia (AA) remains poorly defined. The prognostic value of donor–recipient age difference (DRAD) has rarely been explored.

**Objective:**

To delineate the optimal donor age window and investigate the prognostic significance of the DRAD in AA patients undergoing allo-HSCT.

**Methods:**

This retrospective study included 261 AA patients who received first allo-HSCT between 2018 and 2025. Patients were stratified into four groups based on donor age quartiles (G1: <20 yrs; G2: 20–29 yrs; G3: 29–38 yrs; G4: >38 yrs) and also by DRAD (DRAD1: <-10yrs; DRAD2: -10 to 10yrs; DRAD3: >+10 yrs). Primary endpoints were overall survival (OS), failure-free survival (FFS), and GVHD-free, relapse-free survival (GRFS).

**Results:**

Donor age stratification alone did not significantly affect overall transplant outcomes. However, pairwise comparison showed that the G4 had significantly better OS than the G1 (5-year OS: 88.9% vs. 75.8%; *P* = 0.047). In multivariable Cox regression, donor age was not an independent predictor, but DRAD emerged as a critical factor. Compared with DRAD2 and DRAD3, DRAD1 was associated with significantly inferior neutrophil (*P* = 0.008) and platelet (*P*<0.001) engraftment, overall response (*P* = 0.029), OS (*P*<0.001), FFS (*P*<0.001) and GRFS (*P*=0.001). DRAD3 showed comparable OS, FFS, and GRFS to DRAD2, but had a significantly higher rate of EBV reactivation (74.6% vs. 59.1% and 59.0%, *P* = 0.040), suggesting that age-matched donor-recipient pairing may confer a favorable prognosis.

**Conclusion:**

Donor age alone did not independently predict outcomes, whereas DRAD is a more impactful clinical parameter. A much younger donor (DRAD1) predicts poor outcomes, while age-matched donor-recipient pairing (DRAD2) offers the best balance of efficacy and safety. These findings raise the hypothesis that donor-recipient age matching may be preferable to simply pursuing the youngest donor. However, given the single-center nature of this study, external validation in independent cohorts is warranted before routine adoption in clinical practice.

## Introduction

Aplastic anemia (AA) is an immune-mediated bone marrow failure syndrome characterized by pancytopenia and hypocellular marrow. For patients with severe or very severe AA (SAA/VSAA) who have a human leukocyte antigen (HLA)-matched donor, allogeneic hematopoietic stem cell transplantation (allo-HSCT) remains the only curative treatment (1, 2). Over the past decade, refinements in conditioning regimens, graft-versus-host disease (GVHD) prophylaxis, and supportive care have substantially improved early post-transplant outcomes, with 5-year overall survival (OS) now exceeding 80% in experienced centers (3–5). However, long-term complications, including virus reactivation, GVHD and impaired quality of life, continue to challenge treatment durability and patient well-being (6, 7). Consequently, optimizing donor selection to maximize long-term survival and health-related quality of life has become a pivotal task in contemporary transplant practice for AA.

Donor age is recognized as a key factor influencing allo-HSCT outcomes. The prevailing paradigm posits that younger donors are superior, attributed to better hematopoietic cell function, longer telomere length, more robust immune reconstitution, and a lower risk of complications. This linear “younger-is-better” model, however, is largely derived from studies in hematologic malignancies. In that context, the graft-versus-tumor effect and intensive conditioning regimens may confound or modify the impact of donor age (8–10). Whether this linear model applies directly to AA, a non-malignant disease where the graft-versus-marrow effect is irrelevant, remains uncertain.

AA patients present a unique physiological setting. They typically receive less intensive conditioning to reduce toxicity, yet face a higher risk of graft rejection and delayed immune reconstitution (11, 12). Interestingly, a recent study in malignant diseases revealed a non-linear relationship between donor age and survival, suggesting an “optimal age window” rather than a simple age gradient (13). Whether the impact of donor age follows a similar non-linear pattern in AA is unknown. Furthermore, the interaction between donor and recipient age, the donor-recipient age difference (DRAD), has been scarcely explored. Understanding this interplay could lead to more personalized donor selection, moving beyond a one-size-fits-all approach. Therefore, this study aimed to: (1) characterize the precise relationship between donor age and major post-transplant outcomes in a large AA cohort; (2) identify an optimal donor age window; (3) evaluate the prognostic value of the DRAD.

## Patients and methods

### Study design and ethics

This retrospective study enrolled all consecutive AA patients who underwent first allo-HSCT at our center between January 2018 and June 2025. This study was approved by the Ethics Committee of our hospital (Approval number: 2025-KLS-631-01) and adhered to the Declaration of Helsinki. Informed consent was waived due to the retrospective design.

### Patient population

Inclusion criteria: (1) first allo-HSCT between January 2018 and June 2025, with follow-up more than 6 months; (2) diagnosis of SAA or VSAA according to the Camitta criteria (2); (3) complete clinical data.

### Donor age and DRAD stratification

**Figure 1:**
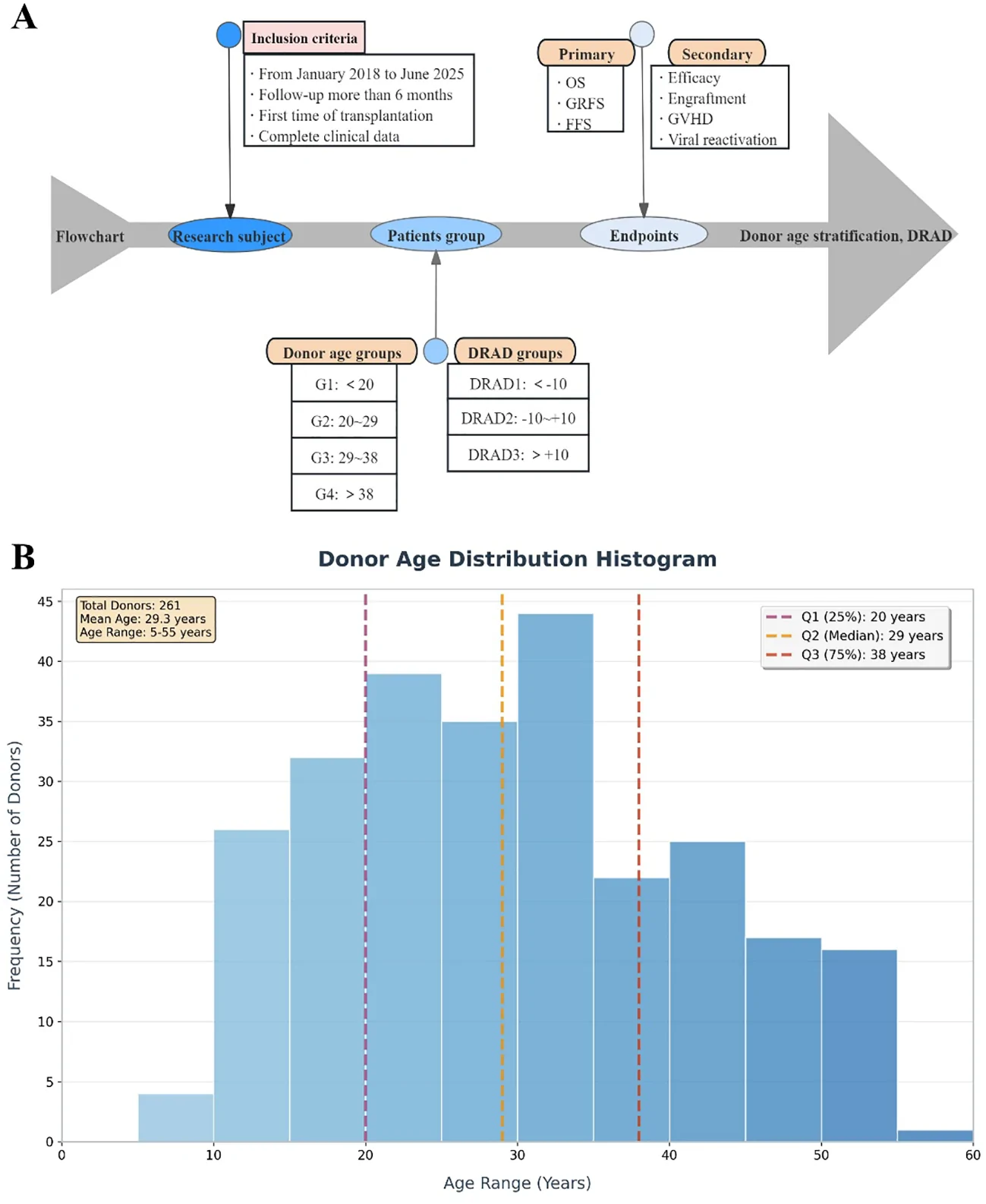
Study flow diagram and donor age distribution. **(A)** Flow diagram showing patient selection process. **(B)** Histogram of donor age with quartile cutoffs indicated by dashed lines.

To assess the impact of donor age, patients were divided into four groups based on donor age quartiles (**Figure 1**): G1, <20 yrs (very young); G2, 20–29 yrs (optimal); G3, 29–38 yrs (middle-aged); G4, >38 yrs (older).

The donor-recipient age difference (DRAD) was calculated as donor age minus recipient age. Based on this continuous variable, patients were categorized into three groups: DRAD1, < -10 yrs (donor significantly younger); DRAD2, -10 to 10 years (age-matched); DRAD3, > +10 years (donor significantly older).

### Transplantation procedures

Conditioning and GVHD prophylaxis regimens include the modified FCA regimen, the classic BuCy regimen, and the novel FABT regimen. Detailed procedures and dosages are described in our previous studies (14, 15). Given the reported role of mesenchymal stem cells (MSC) and umbilical cord blood (UCB) in promoting engraftment and long-term survival (16), we routinely recommend for all patients to improve transplant tolerance. Third-party MSC were infused at a median dose of 1×10^6^ cells/kg on day −1 or day 0. UCB and MSC were used as adjunctive grafts for only once. Indications were physician-directed, primarily for patients receiving haploidentical transplants or those at high risk for engraftment failure/poor graft function.

### Definition and assessment

The primary endpoints were OS, GVHD-free and relapse-free survival (GRFS), and failure-free survival (FFS). Secondary endpoints included engraftment, incidence of acute and chronic GVHD, viral reactivation, and treatment efficacy.

Neutrophil Engraftment: Absolute neutrophil count reaching 0.5×10^9^/L on three consecutive days by day 28 post-transplant. Platelet Engraftment: Platelet count reaching 20×10^9^/L for seven consecutive days without transfusion support by day 28 post-transplant. GVHD: aGVHD diagnosis was based on the modified Glucksberg criteria (17), and cGVHD diagnosis on the 2014 revised NIH consensus criteria (18). Virus reactivation: CMV-DNA copy number exceeding 10^3^ copies/mL or EBV-DNA copy number exceeding 10^4^ copies/mL on two consecutive tests. Overall response (OR): Complete response (CR) + partial response (PR) according to efficacy grading criteria (19). OS: Time from allo-HSCT to death or last follow-up. FFS: Time from allo-HSCT to treatment failure (including death, disease relapse, or failure to achieve OR) or last follow-up. GRFS: Survival without disease relapse, grade III-IV aGVHD, or extensive cGVHD.

### Statistical analysis

Statistical analyses were performed using SPSS version 27.0 (IBM, Armonk, NY) and GraphPad Prism version 8.0 (GraphPad Software, San Diego, CA). Continuous variables that were not normally distributed are presented as median (range) and were compared using the Mann-Whitney U test. Categorical variables are expressed as frequencies and percentages and compared using the chi-square test. Survival analysis was performed using the Kaplan-Meier method, and survival curves were compared using the log-rank test. Cox proportional hazards regression models were used to assess the independent prognostic value of donor age for OS, FFS, and GRFS, adjusting for clinically relevant covariates. Variables with *P* < 0.10 in univariable analysis, as well as clinically established confounders (donor type, conditioning regimen, DSA, HCT-CI), were entered into the multivariable model. Results are reported as hazard ratios (HR) with 95% confidence intervals (CI). A *P*-value of less than 0.05 was considered statistically significant.

## Results

### Patient and transplant characteristics

A total of 261 AA patients were included in the final analysis. The baseline characteristics stratified by the four donor age groups (G1, n=62; G2, n=63; G3, n=64; G4, n=72) are summarized in **Table 1**. Significant differences were observed in donor sex (*P* = 0.015), donor type (*P* < 0.001), and graft source (*P* < 0.001), reflecting clinical practice where older donors are more often male and from haploidentical sources. Recipient characteristics were well balanced across groups.

**Table 1:** Baseline characteristic stratified by donor age groups.

Characteristics	G1 (N = 62)	G2 (N = 63)	G3 (N = 64)	G4 (N = 72)	*P*
Recipient characteristics					
Age, median (range), years	31 (10-52)	31 (7-60)	31 (9-61)	31 (8-66)	0.676
Male, n (%)	30 (48.4)	34 (54.0)	35 (54.7)	36 (50.0)	0.870
Female, n (%)	32 (51.6)	29 (46.0)	29 (45.3)	36 (50.0)	
Diagnosis					0.459
NSAA+SAA, n (%)	34 (54.8)	34 (54.0)	40 (62.5)	47 (65.3)	
VSAA, n (%)	28 (45.2)	29 (46.0)	24 (37.5)	25 (34.7)	
Interval from diagnosis to transplantation					0.634
≤1 year, n (%)	39 (62.9)	40 (63.5)	39 (60.9)	51 (70.8)	
>1 year, n (%)	23 (37.1)	23 (36.5)	25 (39.1)	21 (29.2)	
ABO blood type					0.729
Matched, n (%)	34 (54.8)	30 (47.6)	35 (54.7)	41 (56.9)	
Mismatched, n (%)	28 (45.2)	33 (52.4)	29 (45.3)	31 (43.1)	
HCT-CI					0.527
0, n (%)	44 (71.0)	47 (74.6)	42 (65.6)	55 (76.4)	
>1, n (%)	18 (29.0)	16 (25.4)	22 (34.4)	17 (23.6)	
DSA Positive, n (%)	13 (21.0)	10 (15.9)	9 (14.1)	15 (20.8)	0.653
Pre-transplant					
WBC, median (range), ×10^9^/L	1.7 (0.1-2.9)	1.7 (0.1-2.9)	1.7 (0.3-2.8)	1.8 (0-2.8)	0.604
ANC, median (range), ×10^9^/L	0.4 (0-1.3)	0.4 (0-1.9)	0.4 (0-1.8)	0.4 (0-2.1)	0.437
HGB, median (range), g/L	57 (28-67)	57 (27-70)	54 (14-74)	56 (25-71)	0.114
PLT, median (range), ×10^9^/L	11 (2-38)	12 (1-37)	12 (1-31)	12 (1-31)	0.648
Ferritin, median (range), ng/mL	776 (37-11922)	792 (52-8001)	732 (8-12374)	755 (55-17747)	0.174
Donor characteristics					
Male, n (%)	34 (54.8)	31 (49.2)	43 (67.2)	53 (73.6)	**0.015**
Female, n (%)	28 (45.2)	32 (50.8)	21 (32.8)	19 (26.4)	
Donor type					<0.001
MSD, n (%)	5 (8.1)	6 (9.5)	13 (20.3)	9 (12.5)	
HID, n (%)	57 (91.9)	44 (69.8)	31 (48.4)	58 (80.6)	
MUD, n (%)	0 (0.0)	13 (20.6)	20 (31.3)	5 (6.9)	
Transplant characteristics					
Conditioning regimen					0.869
FABT, n (%)	10 (16.1)	13 (20.6)	14 (21.9)	18 (25.0)	
FCA, n (%)	40 (64.5)	42 (66.7)	40 (62.5)	44 (61.1)	
BuCy, n (%)	12 (19.4)	8 (12.7)	10 (15.6)	10 (13.9)	
Graft source					<0.001
PBSC, n (%)	5 (8.1)	19 (30.2)	25 (39.1)	10 (13.9)	
BMSC+PBSC, n (%)	57 (91.9)	44 (69.8)	39 (60.9)	62 (86.1)	
UCB, n (%)	24 (38.7)	27 (42.9)	36 (56.3)	23 (31.9)	**0.035**
MSC, n (%)	32 (51.6)	20 (31.7)	27 (42.2)	37 (51.4)	**0.074**
CD34+ cell, median (range), ×10^6^/kg	6.00 (1.69-25.38)	5.98 (2.11-19.21)	6.21 (1.95-16.38)	5.66 (2.12-20.01)	0.206

HCT-CI, hematopoietic cell transplantation-specific comorbidity index; DSA, donor-specific antibody; WBC, white blood cell count; ANC, absolute neutrophil count; HGB, hemoglobin; PLT, platelet; MSD, matched sibling donor; HID, haploidentical donor; MUD, matched unrelated donor; PBSC, peripheral blood stem cell; BMSC, bone marrow stem cell; UCB, umbilical cord blood; MSC, mesenchymal stem cell.

Bold values indicate p < 0.05.

### Transplantation outcomes by donor age group

**Figure 2:**
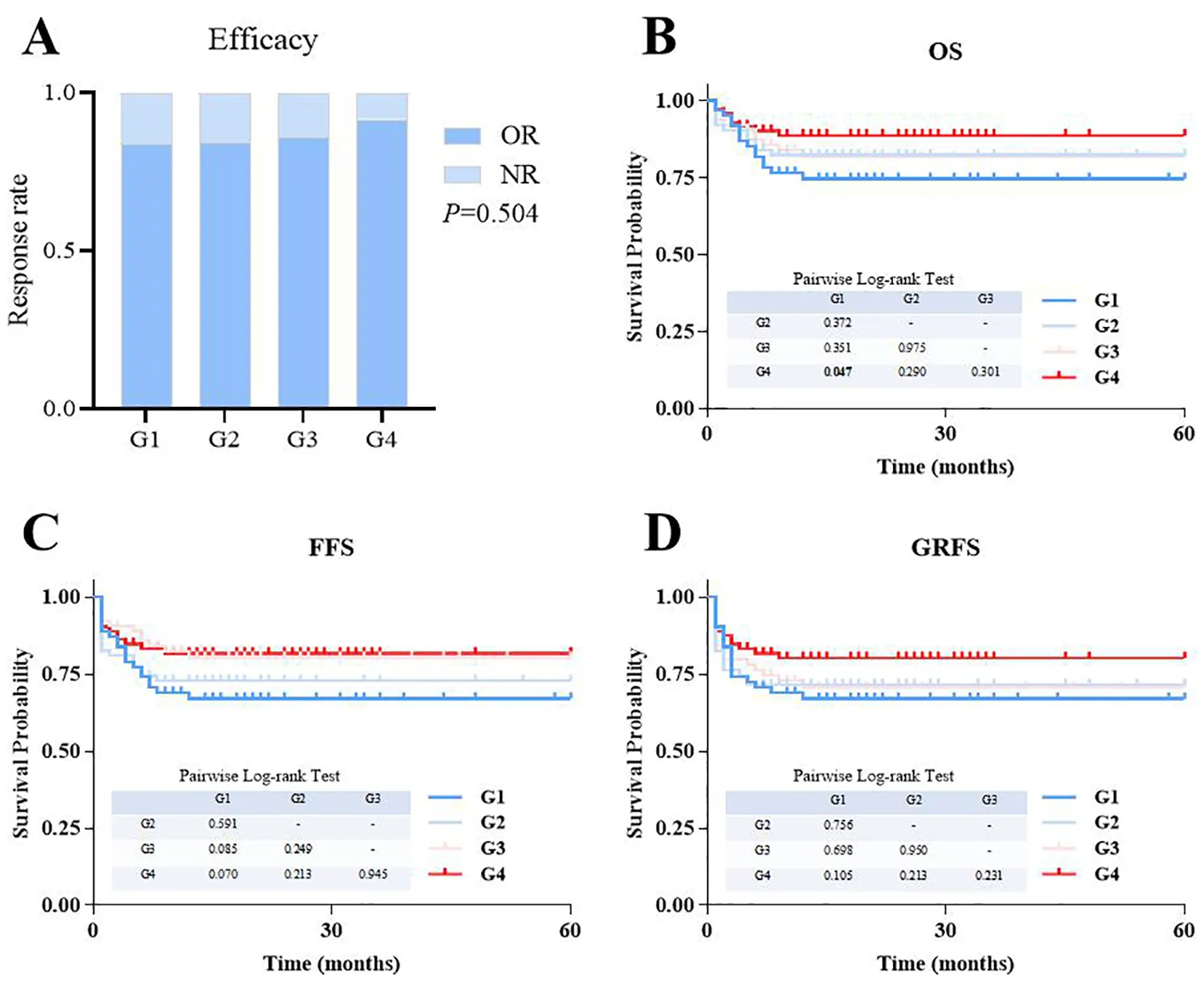
Survival outcomes by donor age groups. **(A)** Efficacy, **(B)** OS, **(C)** FFS, and **(D)** GRFS stratified by donor age quartiles.

The incidences of engraftment, aGVHD, II-IV aGVHD, cGVHD, and viral reactivation were comparable among the four groups. OR rates showed a trend towards improvement with increasing donor age (83.9%, 84.1%, 85.9%, 91.7%) but did not reach statistical significance (*P* = 0.504). Although no significant differences were observed in 5-year OS (75.8%, 82.5%, 82.8%, 88.9%; *P* = 0.284), 5-year FFS (67.7%, 73.0%, 81.3%, 81.9%; *P* = 0.192), or 5-year GRFS (67.7%, 71.4%, 71.9%, 80.6%; *P* = 0.444), the G4 group (donor age >38 yrs) numerically exhibited the highest rates. Detailed survival outcomes are presented in **Table 2** and **Figure 2**.

**Table 2:** Transplantation outcomes by donor age groups.

Endpoint	G1 (N = 62)	G2 (N = 63)	G3 (N = 64)	G4 (N = 72)	*P*
Engraftment					
NE engraftment, n (%)	60 (96.8)	60 (95.2)	60 (93.8)	72 (100.0)	0.220
Time to engraftment, median (range)	13 (9-23)	13 (9-28)	13 (8-20)	13 (8-27)	0.724
PLT engraftment, n (%)	50 (80.6)	51 (81.0)	57 (89.1)	62 (86.1)	0.487
Time to engraftment, median (range)	16 (10-28)	16 (8-28)	16 (8-27)	16 (9-26)	0.220
GVHD					
aGVHD, n (%)	26 (41.9)	26 (41.3)	22 (34.4)	22 (30.6)	0.455
II-IV aGVHD, n (%)	9 (14.5)	11 (17.5)	9 (14.1)	10 (13.9)	0.935
cGVHD, n (%)	16 (25.8)	17 (27.0)	11 (17.2)	15 (20.8)	0.521
Virus reactivation					
EBV, n (%)	39 (62.9)	34 (54.0)	42 (65.6)	49 (68.1)	0.365
CMV, n (%)	26 (41.9)	15 (23.8)	19 (29.7)	20 (27.8)	0.144
Treatment efficacy					0.504
OR, n (%)	52 (83.9)	53 (84.1)	55 (85.9)	66 (91.7)	
NR, n (%)	10 (16.1)	10 (15.9)	9 (14.1)	6 (8.3)	
Survival outcomes					
5-year OS, n (%)	47 (75.8)	52 (82.5)	53 (82.8)	64 (88.9)	0.284
5-year FFS, n (%)	42 (67.7)	46 (73.0)	52 (81.3)	59 (81.9)	0.192
5-year GRFS, n (%)	42 (67.7)	45 (71.4)	46 (71.9)	58 (80.6)	0.444
Follow-up time, median (range)	19 (1-60)	17 (1-60)	16 (1-60)	17 (1-60)	0.295

NE, neutrophil; PLT, platelet; aGVHD, acute graft-versus-host disease; cGVHD, chronic graft-versus-host disease; EBV, Epstein-Barr virus; CMV, cytomegalovirus; OR, overall response; NR, no response; OS, overall survival; FFS, failure-free survival; GRFS, GVHD-free, relapse-free survival.

### Impact of DRAD

We next evaluated the prognostic impact of DRAD. The baseline characteristics stratified by the DRAD (DRAD1, n=98; DRAD2, n=100; DRAD3, n=63) are summarized in **Supplementary Table 1**. Significant differences were observed in recipient diagnosis (*P* = 0.029), HCT-CI (*P* < 0.001), DSA positivity (*P* = 0.003), donor type (*P* < 0.001), conditioning regimen (*P* = 0.025), and the use of UCB (*P* = 0.041). These differences reflect distinct clinical profiles across DRAD groups: DRAD1 was characterized by older recipient age, higher comorbidity burden, and greater proportion of HIDss and DSA positivity; DRAD2 had more MSDs and more favorable HCT-CI scores; DRAD3 showed the youngest recipient age and frequent use of HIDs. Other baseline characteristics, including interval from diagnosis to transplantation, ABO matching, pre-transplant laboratory parameters, and CD34+ cell dose, were comparable among the three groups.

**Figure 3:**
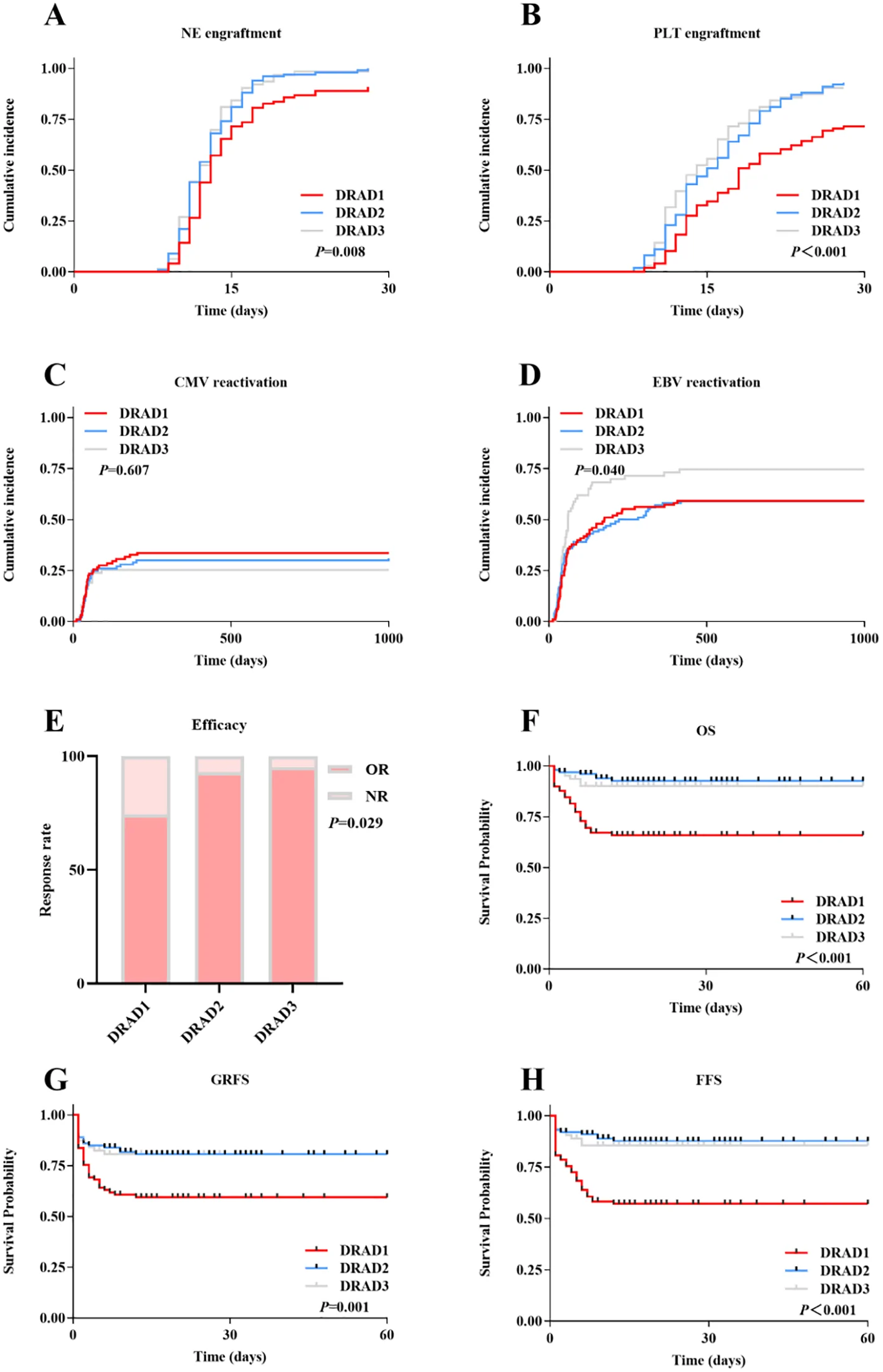
Transplantation outcomes stratified by DRAD. **(A)** NE engraftment. **(B)** PLT engraftment. **(C)** CMV reactivation. **(D)** EBV reactivation. **(E)** Efficacy. **(F)** OS. **(G)** GRFS. **(H)** FFS. P-values were calculated using the log-rank test (for OS and GRFS) or Gray’s test (for cumulative incidence).

As shown in **Figure 3**, the DRAD1 showed significantly inferior engraftment rates compared with DRAD2 and DRAD3 (NE: 90.8% vs. 100% and 100%, *P* = 0.008; PLT: 71.4% vs. 93.0% and 90.4%, *P* < 0.001). While CMV reactivation was comparable across groups (*P* = 0.607), EBV reactivation was markedly higher in the DRAD3 group (74.6% vs. 59.1% and 59.0%, *P* = 0.040). The DRAD1 group had the lowest OR rate of 74.4%, compared with 93.0% in DRAD2 and 95.2% in DRAD3 (*P* = 0.029). Survival outcomes were poorest in DRAD1: 5-year OS (67.3% vs. 93.0% and 90.4%, *P* < 0.001), 5-year GRFS (60.2% vs. 81.0% and 80.9%, *P* = 0.001), and 5-year FFS (58.1% vs. 88.0% and 85.7%, *P* < 0.001) (**Figure 3**).

**Figure 4:**
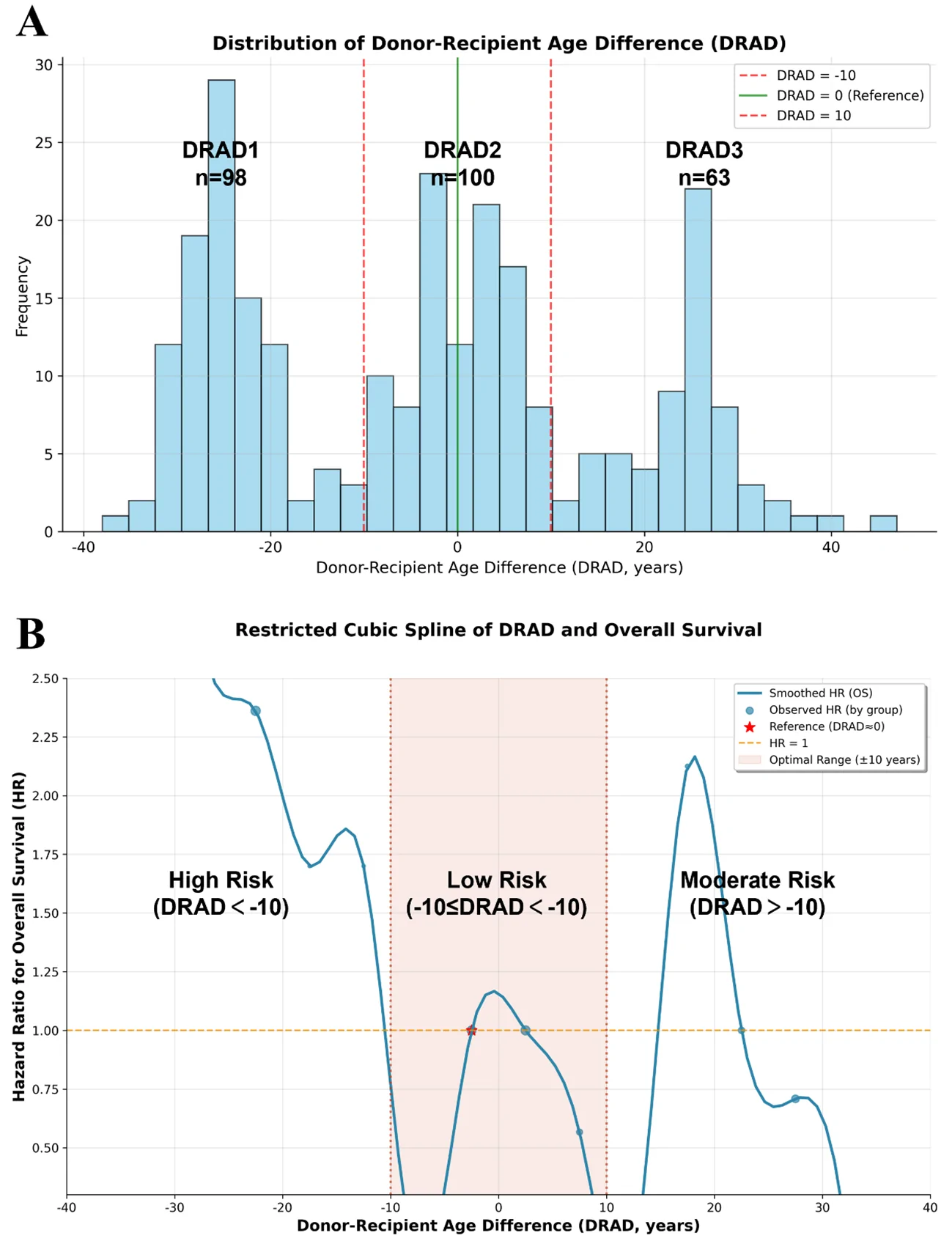
Distribution of DRAD and its association with OS. **(A)** Frequency distribution of DRAD, with markers indicating DRAD = –10, 0 (reference), and 10. **(B)** Relationship between DRAD and HR for OS fitted using restricted cubic splines. Using DRAD = 0 as reference (HR = 1), both the smoothed HR curve (solid line) and the observed HRs by group show that HR approaches 1 when DRAD is within the range of ±10 years, suggesting that patients with an age difference in this interval have a lower risk of overall mortality.

Restricted cubic spline (RCS) analysis further validated that the optimal DRAD window lies within ±10 years (**Figure 4**). Within this interval, the HR for OS was closest to 1, indicating no significant survival risk difference compared with the reference (DRAD = 0). In contrast, DRAD1 was associated with a markedly elevated HR (mean 11.493), and DRAD3 with a moderate increase (mean 2.595). These findings further support the importance of an age-matched donor selection strategy.

### Independent prognostic value and non-linear relationship

Multivariable analysis identified DRAD1 as an independent adverse prognostic factor for OS (HR = 11.884, *P* < 0.001), FFS (HR = 5.632, *P* < 0.001), and GRFS (HR = 2.869, *P* = 0.004) compared with DRAD2, whereas DRAD3 showed no significant difference, indicating a non-linear risk distribution. Conditioning with FCA or BuCy independently predicted worse outcomes relative to FABT. DSA negativity was protective, and grade II–IV aGVHD significantly increased mortality and reduced GRFS. Donor age stratification, donor type, UCB, and MSC were not independently associated with outcomes (**Table 3**).

**Table 3:** Multivariate analysis of the factors associated with OS, FFS, GRFS.

Variable	Comparison	OS		FFS		GRFS	
		HR (95% CI)	P	HR (95% CI)	P	HR (95% CI)	P
Donor age stratification	G2 vs. G1	1.006 (0.415-2.435)	0.990	1.133 (0.551-2.330)	0.734	1.072 (0.511-2.248)	0.855
	G3 vs. G1	1.937 (0.699–5.371)	0.204	1.164 (0.484-2.800)	0.735	1.416 (0.651-3.081)	0.381
	G4 vs. G1	2.934 (0.771–11.168)	0.114	2.807 (0.945-8.338)	0.063	0.946 (0.359-2.492)	0.911
DRAD	DRAD1 vs. DRAD2	11.884 (3.954-35.716)	**<0.001**	5.632 (2.410-13.163)	**<0.001**	2.869 (1.389-5.926)	**0.004**
	DRAD3 vs. DRAD2	0.910 (0.229–3.616)	0.894	0.743 (0.244-2.258)	0.600	1.417 (0.558-3.602)	0.464
Conditioning regimen	FCA vs. FABT	7.589 (2.368–24.329)	**<0.001**	3.838 (1.565-9.412)	**0.003**	4.119 (1.698-9.996)	**0.002**
	BuCy vs. FABT	6.779 (1.781–25.808)	**0.005**	4.128 (1.435-11.873)	**0.009**	3.602 (1.227-10.573)	**0.020**
Donor type	HID vs. MSD	5.933 (0.703–50.050)	0.102	3.360 (0.688-16.416)	0.134	2.172 (0.607-7.773)	0.233
	MUD vs. MSD	2.905 (0.264–31.986)	0.383	3.945 (0.637-24.423)	0.140	1.186 (0.225-6.252)	0.841
Transplant interval	>1 year vs. ≤1 year	1.661 (0.801–3.442)	0.173	0.825 (0.447-1.524)	0.539	1.424 (0.802-2.526)	0.227
Graft source	PB+BM vs. PB	0.320 (0.103–0.990)	**0.048**	0.421 (0.159-1.111)	0.081	0.640 (0.231-1.775)	0.391
UCB	Yes vs. No	0.748 (0.360–1.557)	0.438	0.661 (0.358-1.220)	0.186	1.064 (0.603-1.879)	0.829
MSC	Yes vs. No	0.760 (0.362–1.598)	0.469	1.090 (0.597-1.991)	0.779	1.065 (0.600-1.888)	0.830
DSA	Negative vs. Positive	0.277 (0.111–0.691)	**0.006**	0.451 (0.211-0.966)	**0.040**	0.420 (0.195-0.993)	**0.026**
II-IV aGVHD	Yes vs. No	3.373 (1.515–7.513)	**0.003**	1.739 (0.891-3.397)	0.105	16.638 (9.009-30.726)	<0.001
cGVHD	Yes vs. No	0.301 (0.128–0.705)	**0.006**	0.570 (0.298-1.093)	0.091	0.689 (0.368-1.289)	0.243
CMV	Yes vs. No	0.710 (0.323–1.560)	0.393	1.460 (0.810-2.633)	0.209	0.616 (0.330-1.148)	0.127
EBV	Yes vs. No	0.269 (0.132–0.548)	**<0.001**	0.523 (0.293-0.933)	**0.028**	0.643 (0.361-1.146)	0.134

Bold values indicate p < 0.05.

## Discussion

In this single-center retrospective study of 261 AA patients undergoing allo-HSCT, we have three principal findings. First, donor age stratification alone did not independently predict survival, prompting reconsideration of the unqualified emphasis on donor youth. Second, DRAD emerged as a more impactful parameter: patients with a much younger donor (DRAD <-10 years, DRAD1) experienced significantly inferior engraftment, response, and survival. Third, donor-recipient age matching within ±10 years (DRAD2) conferred the best prognosis, with a 5-year OS of 93.0% versus 67.3% in DRAD1 and 90.4% in DRAD3 (*P* < 0.001), and comparable GRFS while avoiding the excess EBV reactivation seen in DRAD3.

The biological interpretations presented below are speculative hypotheses drawn from existing literature and require direct mechanistic validation; notably, our retrospective dataset did not include immune reconstitution kinetics, T-cell subset analyses, telomere measurements, or CHIP status. We hypothesize that the poor outcomes in DRAD1 might partly reflect the immunological immaturity of very young donors, characterized by a higher proportion of naïve T cells (20), reduced regulatory T-cell function (21), and delayed post-transplant immune reconstitution (22). These features increase risks of graft rejection, infection, and potentially poor graft function. In our data, DRAD1 showed the lowest neutrophil (**Figure 3A**) and platelet engraftment (**Figure 3B**), consistent with delayed or failed engraftment due to inadequate immune priming. Specifically, naïve T cells require thymic-dependent maturation pathways that may be insufficient in the immediate post-transplant period, leading to compromised hematopoietic support. This pattern aligns with observations in umbilical cord blood transplantation, where the predominance of antigen-inexperienced naïve T cells contributes to profound early T-cell lymphopenia and impaired functional immunity (23). Notably, this pattern also mirrors recent observations in malignant diseases where the youngest donors failed to show superior outcomes (24), suggesting that immunological immaturity could, in theory, offset the theoretical advantages of younger stem cells, though this remains to be tested with mechanistic data.

The optimal outcomes observed in the DRAD2 define a “Goldilocks zone” for donor selection. Within this window, the donor immune system is sufficiently mature to provide effective hematopoietic support and pathogen protection, yet not senescent to provoke excessive GVHD or impair engraftment. DRAD2 patients achieved the highest 5-year OS and FFS, with significantly lower EBV reactivation than DRAD3 (59.1% vs. 74.6%, *P* = 0.040). We speculate that age-matched donors may offer a more favorable balance of telomere length, T-cell receptor repertoire diversity, and hematopoietic stem cell clonal dynamics. If this hypothesis holds, such a balance could facilitate more robust immune reconstitution. However, our clinical data cannot directly confirm these molecular or cellular mechanisms.

The impact of donor age in AA differs fundamentally from that in hematologic malignancies such as AML and MDS. In malignant diseases, recent large-scale registry studies have consistently demonstrated that younger donors confer superior outcomes, primarily through enhanced graft-versus-tumor (GVT) effects. For instance, in older AML patients, allo-HCT from younger matched unrelated donors (age ≤35 years) was associated with significantly reduced relapse risk compared to older matched sibling donors (age ≥50 years), translating into improved disease-free survival (25, 26). Similarly, in MDS, donor age interacts with donor type to shape outcomes, with young mismatched donors achieving survival comparable to matched donors, while old mismatched donors show inferior outcomes (27, 28). The disparate findings between AA and malignant diseases likely stem from the presence or absence of GVT effects. In AA, a non-malignant condition where the graft-versus-marrow effect is pathophysiologically irrelevant, the benefits of younger donors’ robust immune surveillance against tumor cells are absent (1). Instead, the disadvantages of immunological immaturity (in very young donors) and cellular senescence (in older donors) become more prominent determinants of outcome. This explains why the relationship between donor age and survival follows a non-linear pattern in AA, contrasting with the more linear “younger-is-better” paradigm observed in malignancies where GVT effects dominate (29, 30).

While DRAD is mathematically derived from donor and recipient age, it serves as a clinically pragmatic composite index that captures the immunological mismatch in hematopoietic and immune aging between donor and recipient. Its prognostic value may reflect synergistic effects (e.g., thymic involution in older recipients paired with naïve T-cell predominance in very young donors) that neither age alone captures. For older donors (DRAD3), potential concerns include accumulated cellular senescence, increased burden of clonal hematopoiesis of indeterminate potential (CHIP), and a pro-inflammatory micro-environment that may exacerbate cGVHD risk (31, 32). CHIP, characterized by age-acquired somatic mutations in hematopoietic stem cells, increases exponentially with age and has been associated with donor-derived leukemia and impaired transplant outcomes. Although OS and FFS were comparable to DRAD2 in multivariable analysis, the significantly higher EBV reactivation (74.6% vs. 59.0%, *P* = 0.040) indicates impaired viral-specific immune control. The interaction between donor and recipient age is particularly relevant when a young recipient receives an older donor, as thymic function matching may be suboptimal. We acknowledge baseline imbalances across DRAD groups in diagnosis, HCT-CI, DSA, donor type, conditioning regimen, and UCB use, reflecting real-world donor-selection practices. However, after comprehensive adjustment for these confounders in multivariable Cox regression, DRAD1 remained an independent adverse predictor of OS (HR 11.884, *P* < 0.001), FFS (HR 5.632, *P* < 0.001), and GRFS (HR 2.869, *P* = 0.004), indicating that the observed DRAD effect is unlikely to be fully explained by baseline inter-group differences.

These findings may carry immediate clinical implications for donor selection in AA. When multiple HLA-matched donors are available, we propose prioritizing donors whose age is within ±10 years of the recipient (DRAD2), rather than reflexively selecting the youngest donor. However, we emphasize that HLA matching remains the paramount consideration in donor selection; age matching should serve as a secondary refinement only when HLA compatibility is equivalent among potential donors. Donors aged <20 years (often leading to DRAD1) should be considered relatively sub-optimal, especially for older recipients. Donors aged >38 years (potentially DRAD3) may be acceptable but warrant enhanced EBV monitoring and possibly intensified GVHD prophylaxis, particularly when the recipient is young.

This study is limited by its single-center, retrospective design with inherent selection bias. External validation in an independent, preferably multi-center, cohort is essential before these findings can be translated into clinical practice guidelines. We lacked mechanistic biomarkers such as donor telomere length, T-cell receptor diversity, or CHIP status to directly test the hypothesized biological pathways. Future prospective studies should incorporate biological correlatives to elucidate mechanisms underlying the DRAD effect.

## Conclusion

This study provides hypothesis-generating evidence for a non-linear relationship between donor age and survival after allo-HSCT for AA. Donor-recipient age matching within ±10 years may offer a favorable balance of efficacy and safety, whereas selecting a much younger donor (DRAD1) was associated with unexpectedly poor outcomes. These observations require confirmation in independent, prospective, multi-center cohorts.

## Data Availability

The original contributions presented in the study are included in the article/**Supplementary Material**. Further inquiries can be directed to the corresponding authors.
